# Ultra High Electrical Performance of Nano Nickel Oxide and Polyaniline Composite Materials

**DOI:** 10.3390/polym9070288

**Published:** 2017-07-20

**Authors:** Xiaomin Cai, Xiuguo Cui, Lei Zu, You Zhang, Xing Gao, Huiqin Lian, Yang Liu, Xiaodong Wang

**Affiliations:** 1 College of Material Science and Engineering, Beijing Institute of Petrochemical Technology, Beijing 102617, China; caixiaomin@bipt.edu.cn (X.C.); zulei@bipt.edu.cn (L.Z.); zhangyouwangpai@126.com (Y.Z.); 13552487385@163.com (X.G.); liuyang@bipt.edu.cn (Y.L.); 2 State Key Laboratory of Organic–Inorganic Composites, Beijing University of Chemical Technology, Beijing 100029, China; wangxd@buct.edu.cn; 3 Beijing Key Laboratory of Specialty Elastomer Composite Materials, Beijing Institute of Petrochemical Technology, Beijing 102617, China

**Keywords:** nano nickel oxide, polyaniline, redox electrolyte, supercapacitor

## Abstract

The cooperative effects between the PANI (polyaniline)/nano-NiO (nano nickel oxide) composite electrode material and redox electrolytes (potassium iodide, KI) for supercapacitor applications was firstly discussed in this article, providing a novel method to prepare nano-NiO by using β-cyelodextrin (β-CD) as the template agent. The experimental results revealed that the composite electrode processed a high specific capacitance (2122.75 F·g^−1^ at 0.1 A·g^−1^ in 0.05 M KI electrolyte solution), superior energy density (64.05 Wh·kg^−1^ at 0.2 A·g^−1^ in the two-electrode system) and excellent cycle performance (86% capacitance retention after 1000 cycles at 1.5 A·g^−1^). All those ultra-high electrical performances owe to the KI active material in the electrolyte and the PANI coated nano-NiO structure.

## 1. Introduction

Supercapacitors, with huge potential applications in various electronic devices and electric vehicles, benefit from the advantages of good power density, good cycling life and rapid charge/discharge rate [1,2,3,4]. To further improve the development of supercapacitors, electrode materials that closely affect the properties of supercapacitors should be considered [5,6]. However, Ni based transition metal oxide with remarkable electrochemical properties has become a new kind of energy-storing material [7]. The polyhydroxy structure of β-cyelodextrin (β-CD) can effectively regulate the compatibility with the inorganic compound by the hydrogen bond, using it as the addition of template agent, and a high specific surface area of nano materials can be prepared [8]. This article selected the β-CD as the template agent compounding the precursor at a room temperature, undergoing the hydrothermal modification treatment to get a well dispersive powder of Nano nickel oxide ultimately, which is based on the traditional hydrothermal method. The hydrothermal modification treatment proceeds in the autoclaves with a lining of polytef, using the strong reaction and high dispersing ability of high temperature aqueous system, called “wet” heating process [9]. NiO possesses a disordered internal structure that causes the non-flowing of current carrier; even with a product that has a higher specific surface area using the above method, the defects of poor conductor, narrow working window and low energy density remain the same when NiO is used as a single electrode [10,11,12].

Conducting polymers are widely used as electrochemical [13] active materials for supercapacitors due to their high electrical conductivity and fast charge/discharge kinetics [14]. The charge and discharge process of the conducting polymers is a highly reversible redox reaction, which is rapidly converted between the p-type and n-type doping states, so that the charge is presented not only on the surface but also on the entire volume of the polymer [15,16,17]. Among the various conductive polymers, polyaniline (PANI) is one of the most perspective electrode materials benefited by its lower cost, better conductivity, and higher specific capacitance [15,16,17]. However, when PANI is used in supercapacitors, it suffers from some shortcomings of relatively low electrical conductivity and volume deformation when doping/de-doping of dopants during charging and discharging processes [18,19,20]. In addition, PANI has a poor reversibility compared to carbon and transition metal oxides, and cyclic voltammetry is not an ideal rectangle [18,19,20].

Electro-conductive polymers and nanostructured transition metal oxides provide additional pseudo-capacitances emitted from the Faradaic surface redox [21]. Therefore, the author wants to improve the above questions through the preparation of PANI/nano-NiO composite electrode. Firstly, it should be noted that the nano-NiO were used with alkaline electrolyte system, and the conductivity of PANI can be enhanced in acidic electrolyte. To resolve the conflict, the PANI coated structure was prepared by controlling the proportion of nano-NiO, making sure that most of the nano-NiO was not dissolved in acidic electrolyte under the PANI protection. Furthermore, putting the active material KI into the electrolyte provided the oxide material to make sure the oxidation-reduction reactions of NiO dissolved in acid can take place. The results of scanning electron microscope, X-ray diffractometry, infrared spectroscopy revealed that the formation of PANI chains in the surface of nano-NiO by adsorbing and depositing, that is to say the nano-NiO was coated by PANI. In the three electrode system, an extremely broad working potential window ranged from −0.3 to 0.7 V in 1 M aqueous sulfuric acid and potassium hydroxide. A specific capacitance as high as 2122.75 F·g^−1^ was obtained at a constant current density of 0.1 A·g^−1^. More importantly, the cycling stability of the PANI/nano-NiO was great with a 14% tiny decay in specific capacitance after 1000 cycles at 1.5 A·g^−1^. In addition to the electrode materials, the electrolyte is also a significant factor which affect the performance of supercapacitors. Furthermore, once the potassium iodide (KI) was added to the electrolyte the electrochemical properties of supercapacitors would be increased [22,23].

## 2. Experimental

### 2.1. Chemicals

β-cyelodextrin (β-CD, Aladdin), urea (H_2_NCONH_2_, Aladdin), nickel nitrate hexahydrate ((Ni(NO_3_)_2_∙6H_2_O), Sinopharm Chemical Reagent Co., Ltd. (Beijing, China)), sulfuric acid (H_2_SO_4_, Aladdin (Shanghai, China)), potassium hydroxide (KOH, Aladdin), potassium iodide (KI, Aladdin), hydrochloric acid (HCl, Aladdin), ammonium persulfate (APS, Aladdin), aniline (AN, Aladdin), acetylene black, polyvinylidene fluoride (PVDF, Aladdin), and 1-methyl-2-pyrrolidinone (NMP, Aladdin) were used.

### 2.2. Synthesis

**Figure 1:**
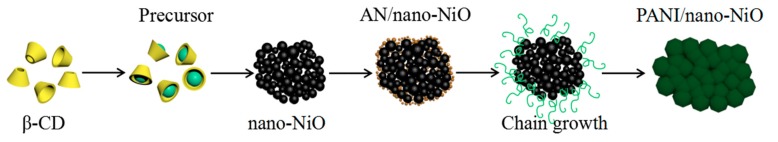
Mimic diagram of synthesis.

The preparation method of PANI/nano-NiO is shown in Figure 1, which relates to the following two parts.

#### 2.2.1. Nanometer Nickel Oxide (nano-NiO) Preparation.

Use the hydrothermal method with β-CD as a template to complete the nano-NiO preparation. Firstly, 5 g of β-CD was dissolved in 100 mL of water at 60 °C. Then, the molar ratio of Ni(NO_3_)_2_∙6H_2_O to urea 4:1 were added to the mixture and ultrasonically dispersed until the solution became transparent. Note that the concentration of metal ions should be controlled in the range of 0.2 mol·L^−1^. The solution was poured into the autoclaves with a lining of polytef and placed in the oven at 140 °C for 24 h. The nano-NiO precursor was washed several times with water and EtOH, dried at 60 °C for 20 h, followed by calcined at 350 °C for 2 h.

#### 2.2.2. PANI/nano-NiO Composites Preparation

Nano-NiO was added to a mixture of AN (5 mL), HCl aqueous (6 mol·L^−1^, 10 mL) and water (100 mL). The mixture was then stirred at 0 °C for 30 min to ensure a stable solution of aniline salt. A mixed solution of APS (11.418 g) and water (50 mL) was added dropwise to the solution of AN salt. Meanwhile the dropping speed should be controlled very slowly allowing the procedure to take 30 min. The reaction was carried out at 0 °C for 3 h. The product was washed several times with water, acetone and diluted HCl, and then dried at 100 °C for 24 h.

#### 2.2.3. Electrode Slices Preparation

PANI/nano-NiO, polyvinylidene fluoride and carbon black in a weight ratio of 8:1:1 were mixed with a few drops of 1-methyl-2-pyrrolidinone (NMP); specifically, the solvent dosage depends on its mixed powder counterpart. The black mixture was ground until it became viscous. Then, the mixture was coated on a stainless steel sheet. Guarantee that the area of sample coating is roughly 1 cm^2^ [24]. Finally dried at 60 °C for 12 h.

In our experiments, the stainless steel material with good corrosion resistance and excellent mechanical properties was used instead of the traditional graphite or nickel foam. The specially pre-treated stainless steel sheet reduces the interface resistance and increases the adhesion to the active material [25,26].

### 2.3. Electrochemical Measurements

The electrochemical measurements were performed by a standard three-electrode cell including the counter electrode (1 cm^2^ Pt foil electrode), the reference electrode (saturated calomel electrode, SCE) and the working electrode. The electrolyte was composed of various concentrations of KI in the acidic or basic aqueous solutions. The galvanostatic charge/discharge analyses were accomplished in the potential range of 0–0.4 V at 0.1, 0.2, 0.5, 1 and 2 A·g^−1^, and the cyclic voltammetry (CV) tests were carried out between −0.3 and 0.7 V at different scan rates [27].

The asymmetric supercapacitor was discussed in a two-electrode system with PANI/nano-NiO as the positive electrode and AC as the negative electrode. The electrochemical measurement of Galvanostatic charge–discharge was carried out in 0.05 M KI solution at room temperature.

### 2.4. Characterization

Scanning electron microscopy (SEM) was carried out in a COXEM-20 microscope (COXEM, Daejeon, Korea) at 20 kV. Infrared spectrometry (IR) analyses were performed on a Thermal Nicolet infrared spectrometer (Thermo Fisher Scientific Inc., Waltham, MA, USA). Wide angle X-ray diffraction (XRD) patterns were obtained with a Bruker D8 diffractometer in reflection mode using Cu Ka = 0.154 nm with a voltage of 40 kV (BRUKER AXS, Berlin, Germany). Thermo gravimetric analyses (TGA) were performed using a TA instruments TGA 2050 from room temperature to 800 °C, with a heating rate of 20 °C min^−1^ under nitrogen.

## 3. Results and Discussion

### 3.1. SEM Analysis

**Figure 2:**
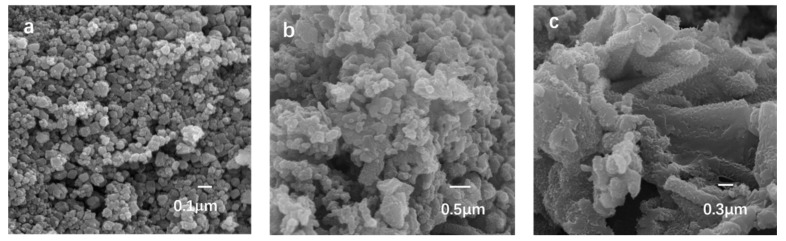
SEM images of: (**a**) nano-NiO; (**b**) PANI/nano-NiO; and (**c**) bulk PANI.

As can be seen clearly in Figure 2a, the nano-NiO possesses uniform particle morphology with a grain size of about 10 nm, consistent with the results of the Scherrer formula [28]. The high surface energy of nanocrystals can lead to a certain degree of reunion. While β-CD as the addition of template agent can greatly reduce the degree of agglomeration and make the product has a good dispersion. On the other hand, the polyhydroxy groups on β-CD can be absorbed on some specific crystal faces in the form of hydrogen bond or process an in-situ complex reaction with crystal nucleus, so that further reunion and NiHCO_3_ nucleus formation will be prevented successfully. Presented in Figure 2c under the effect of the proton acid doping into the PANI enhances the polarity of the molecules and the inter action of molecular chains, which causes the obvious reunion. However, it should be noted that the surface of the nanocomposite becomes loose and the block reunion of bluk PANI is significantly weakened because of the intervention of the nano-NiO (Figure 2b). The crystalline region penetrates into the amorphous region in the overlay area. Thus, it is reasonable to conclude that the nano-NiO was coated by PANI and the nanocrystals play a good supporting role, which can improve the dense and fragile structure of PANI.

### 3.2. FTIR Analysis

**Figure 3:**
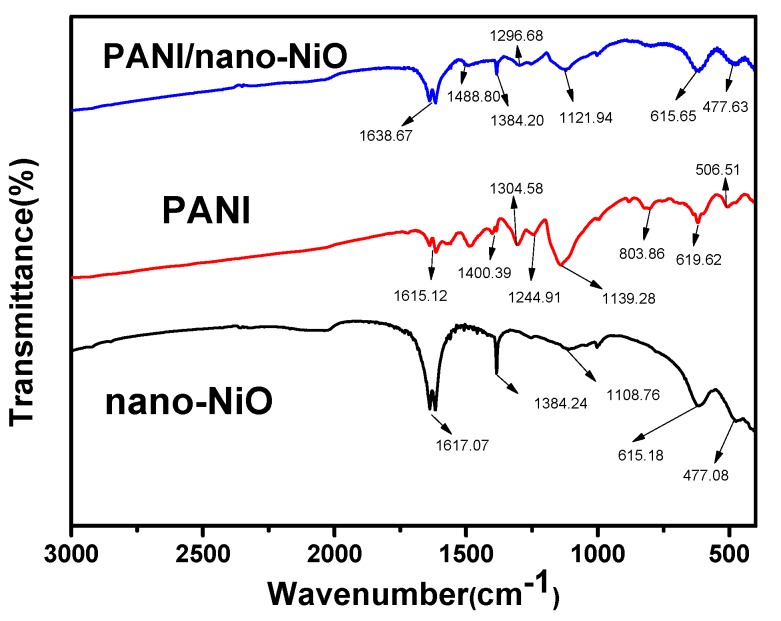
FTIR spectra of nano-NiO, PANI and PANI/nano-NiO.

The FTIR (Fourier Transform Infrared Spectroscopy) spectrum of nano-NiO, PANI and PANI/nano-NiO are shown in Figure 3. The characteristic IR peak of NiO can be attributed to the stretching vibration peak of Ni-O (477.08 cm^−1^) [29], which can also be observed in the PANI/nano-NiO composites emerging at 477.63 cm^−1^. The broad absorption band of at 1400–1500 cm^−1^ [28] is ascribed to the lattice vibration of CO_3_^2−^ which does not appear in NiO, indicating that the nickel oxide was successfully obtained by calcining the precursor. It is obvious that the characteristic peaks of quinoid structure of PANI, C=C stretching vibration of the benzene ring (1615.12 and 1400.39 cm^−1^) and C=N stretching vibration of the benzene ring (1244.91 cm^−1^), all blue shift to higher wave numbers in the PANI/nano-NiO to give stretches at 1638.67, 1488.80, and 1296.68 cm^−1^, respectively, implying a very strong interaction is existed between the PANI and NiO [30]. In addition, the peak of halogen compound at 803.86 to 506.51 cm^−1^ implies that the hydrochloric acid dopes into the PANI and PANI/nano-NiO [31]. This structure gives products an excellent electrical conductivity.

### 3.3. XRD Analysis

**Figure 4:**
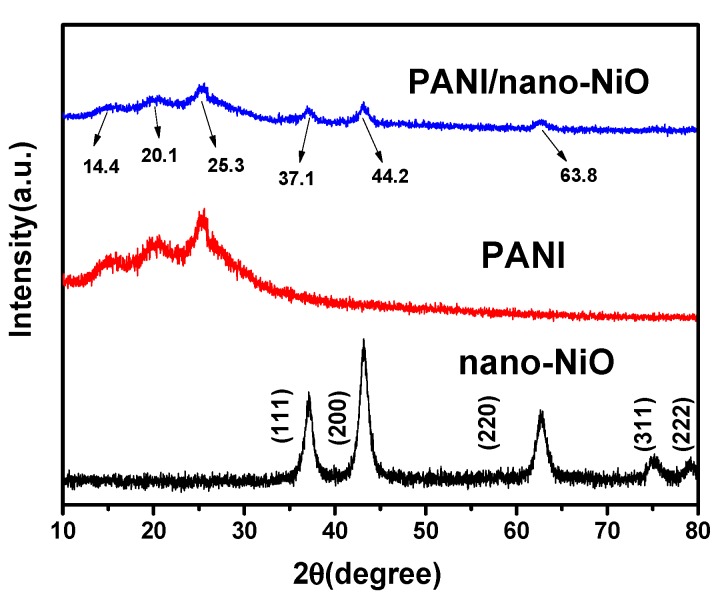
XRD spectra of nano-NiO, PANI, PANI/nano-NiO.

In Figure 4, nano-NiO has five diffraction peaks in the XRD spectra at 36.96° (111), 43.08° (200), 62.56° (220), 74.92° (311) and 78.72° (222), conforming to standard diffraction peak (JCPDS: 78-0643) and these characteristic diffraction peaks appeare clearly in PANI/nano-NiO, which suggests that the nano-NiO has been embedded in the PANI by polymerization reaction, and the nanocomposites still maintain a good crystallization. It is noteworthy that the diffraction peaks of PANI/nano-NiO do not change significantly, but only the diffraction intensity is lower than NiO. This result could be attributed to a relatively low scattering contrast of NiO (about 9.195996 nm in size according to the Scherrer formula [29]) resulting from the formation of PANI chains on the surface of nano-NiO by adsorption and deposition. In other words, it is reasonable to presume that the nano-NiO is coated by PANI.

### 3.4. TGA Analysis

**Figure 5:**
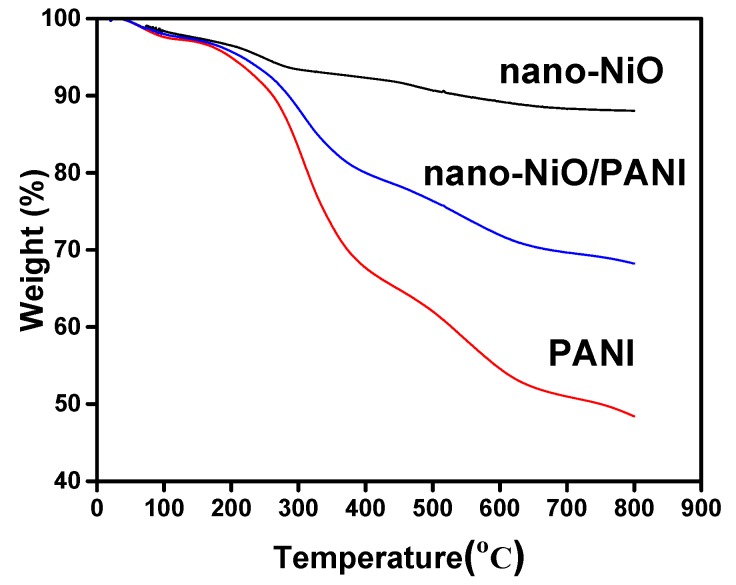
TGA analysis of PANI and PANI/nano-NiO.

Good heat resistance is one of the important indexes to measure the stability of polymers. A characteristics two-step process of PANI's lapse is exhibited in Figure 5 (one corresponds to do-pant molecules in the temperature range from 200 to 300 °C and the other is the elimination of PANI degradation). When the temperature comes up to 800 °C, there is about 48% of PANI left, which is attributed to the carbonization in nitrogen atmosphere. Moreover, it is also noteworthy that the total weight loss of PANI/nano-NiO is about 75%. Clearly, the thermal weight loss of PANI/nano-NiO is less than that of PANI, which means the composite possesses better stability.

### 3.5. Electrochemical Properties Analysis

#### 3.5.1. CV Analysis

**Figure 6:**
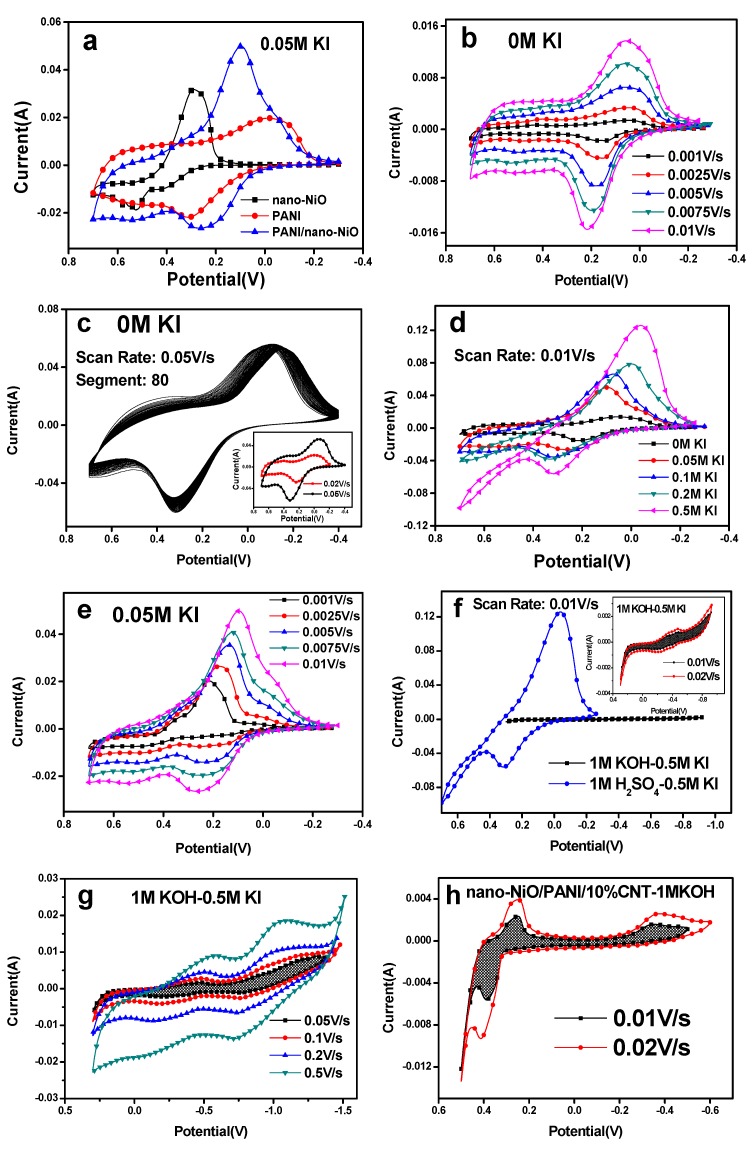
CV curves in different electrolytes, scan rates and concentration of KI. (**a**) 0.05 M KI, 0.01 V/s. (**b**) 0 M KI, 0.001–0.01 V/s. (**c**) 0 M KI, 0.05 V/s. (**d**) 0–0.5 M KI, 0.01 V/s. (**e**) 0.05 M KI, 0.001–0.1 V/s. (**f**) 0.5 M KI, 0.01 V/s. (**g**) 0.5 M KI, 0.05–0.5V/s. (**h**) 0 M KI, 0.01 and 0.02 V/s.

The nanocomposite materials of PANI/nano-NiO are expected to show outstanding performance as electrode material for supercapacitor. The cyclic voltammograms of nano-NiO, PANI and PANI/nano-NiO with a potential window of −0.3 to 0.7 V are shown in Figure 6a, taken at a scan rate of 10 mV·s^−1^, and performed in a mixture of sulfuric acid and 0.05 M KI aqueous. Compared with nano-NiO, the CV loop of PANI/nano-NiO nanocomposite exhibits a much larger integral area and symmetric redox reaction peaks with a clear representation of excellent electrochemical properties. 

Some studies have shown that the redox reaction between I^−^ and IOx^−^ could occur in KI-KOH system [32]. However, in our experiments, the redox peaks can only be seen under high concentrations of KI and scan rate in KI-KOH alkaline system (Figure 6f,g), and even with 10% carbon nanotubes, the CV loops are also much smaller than acidic system (Figure 6h). Furthermore, the oxidation and reduction peaks of acidic system, even after 80 times scanning, are symmetric and no position excursion, which means the products have excellent stability (Figure 6b,c).

To learn more about the role of KI, a series of CV curves of the PANI/nano-NiO in different concentration KI aqueous are shown in Figure 6d. These CV curves possess the obvious redox peaks owing to the oxidation and reduction of PANI. The area of each CV curve was larger than that of lower KI concentration, suggesting a larger capacitance. The higher concentration of KI can provide more I^−^ to combine with the PANI and occur more reversible oxidation-reduction reactions, leading to the increasing of the specific capacitance. Besides, compared with Figure 6b, the vertical axis “Current (A)” has an increased order of magnitude which is also contributed by KI.

As described in Figure 6e, with the increase of the scan rates (range from 0.001 to 0.01 V·s^−1^), the current response of the redox peaks enhances and the oxidation peaks have a tendency to the direction of positive voltage while the reduction peaks have a negative shift [33]. That is to say the redox current increased with increasing scan rate, indicating a good rate capability. The electron exchange occurs between the transform of I_2_, I^−^, I_3_^−^, I_5_^−^ state providing the pseudo capacitance. In conclusion, the chemical reaction mechanism could be proposed as followings (Equations (1)–(6)), comparing with the surface faradic reaction of NiO based electrode in an alkaline electrolyte (Equation (7)) [34]:

(1) $$3I^{-}↔I_{3}^{-}+2e^{-}I_{3}^{-}↔I^{-}+I_{2}\;$$

(2) $$5I^{-}↔I_{5}^{-}+4e^{-}I_{5}^{-}↔I_{3}^{-}+I_{2}$$

(3) $$\mathrm{NiO}+2H^{+}↔{\mathrm{Ni}}^{2+}+H_{2}O$$

(4) $${\mathrm{Ni}}^{2+}+I_{2}+3H_{2}O↔{\mathrm{Ni}}^{3+}+{\mathrm{IO}}_{3}^{-}+I^{-}+6H^{+}+5e^{-}$$

(5) $${\mathrm{Ni}}^{2+}+I_{2}+2H_{2}O↔{\mathrm{Ni}}^{3+}+{\mathrm{IO}}_{2}^{-}+I^{-}+4H^{+}+3e^{-}$$

(6) $${\mathrm{Ni}}^{2+}+I_{2}+H_{2}O↔{\mathrm{Ni}}^{3+}+{\mathrm{IO}}^{-}+I^{-}+2H^{+}+e^{-}$$

(7) $$\mathrm{NiO}+{\mathrm{OH}}^{-}↔\mathrm{NiOOH}+e^{-}$$

#### 3.5.2. GCD Analysis

**Figure 7:**
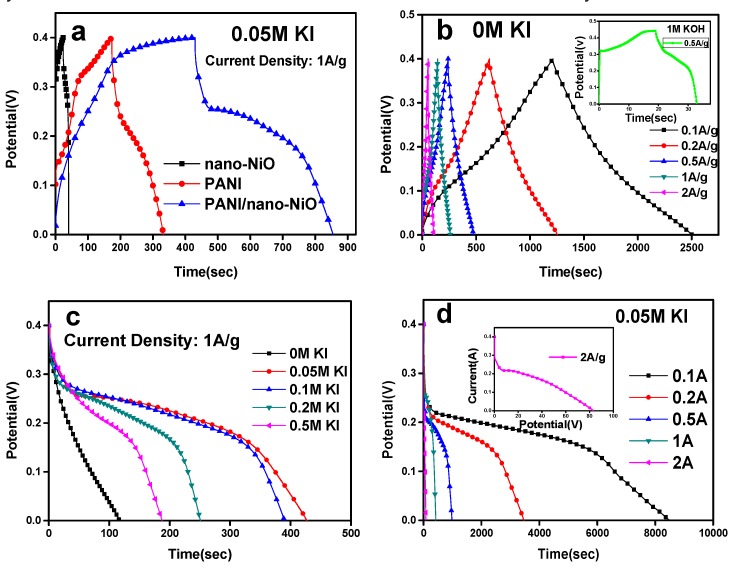
GCD curves in different current densities, electrolytes and concentration of KI. (**a**) 0.05 M KI, 1 A/g. (**b**) 0 M KI, 0.1–2 A/g. (**c**) 0–0.5 M KI, 1 A/g. (**d**) 0.05 M KI, 0.1–2 A/g.

In Figure 7a, compared with nano-NiO and PANI monomer electrodes, the discharge time of composite electrode is greatly enhanced 0.05 M KI at 1 A·g^−1^. As shown in Figure 7b, GCD curves appear in a very symmetrical triangular form, with almost equivalent charge–discharge times, and the discharge time double increases with increasing current density, showing a very good multiplier power characteristic.

When the KI is mixed, the redox reaction of I^−^ provides more pseudo capacitance for the supercapacitor while the discharge time is greatly improved. The composite electrode realizes a maximum discharge time and a largest specific capacity in 0.05 M KI (Figure 7c). Hence, further research proceeds under different current densities in 0.05 M KI (Figure 7d) and the discharge time successively reduces in the wake of the enhancement of the current density.

The specific capacitance (*Cs*, F·g^−1^) and energy density (*E*, Wh·kg^−1^) were calculated based on the following Formulas (8) and (9) [35]:

(8) $$Cs=\frac{It}{m\Delta V}$$

(9) $$E=\frac{1000Cs\Delta V^{2}}{7200}$$

where *I* (A) refers the discharge current, *t* (s) denotes the discharge time, Δ*V* (V) is the potential change during discharge process and *m* (g) is the mass of the active material within the working electrode. All specific capacitance data are collected in Table 1. The results expound that the composite has excellent rate capability.

**Figure 8:**
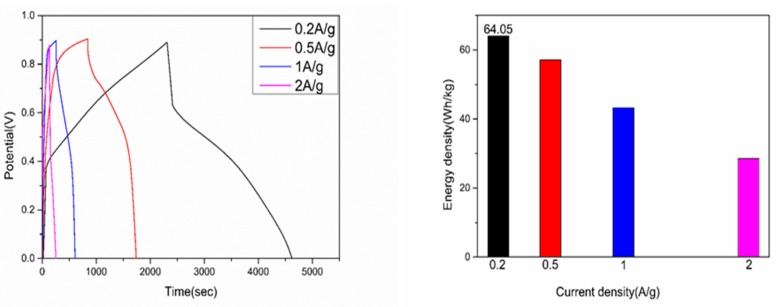
Two-electrode system GCD curves of PANI/nano-NiO at different current density in 0.05 M KI electrolyte and the corresponding energy density.

Furthermore, as depicted in Figure 8, the composite shows a maximum energy density (64.05 Wh·kg^−1^) at 0.2 A·g^−1^ within the potential range from 0 to 0.9 V in the asymmetric two-electrode system assembled by utilizing PANI/nano-NiO as the positive electrode and AC as the negative electrode.

The comparison of the specific capacitance and cycle retention between the system of our present works and the reported values is listed in Table 2. The PANI/nano-NiO composite successfully increases the specific capacitance, energy density (higher than that of pure PANI (41.5 Wh/kg) [3] and NiO (20.14 Wh/kg) [1]) and cycle stability. Overall, it suggests that PANI/nano-NiO have many advantages among many materials from various strategies.

#### 3.5.3. EIS Analysis

**Figure 9:**
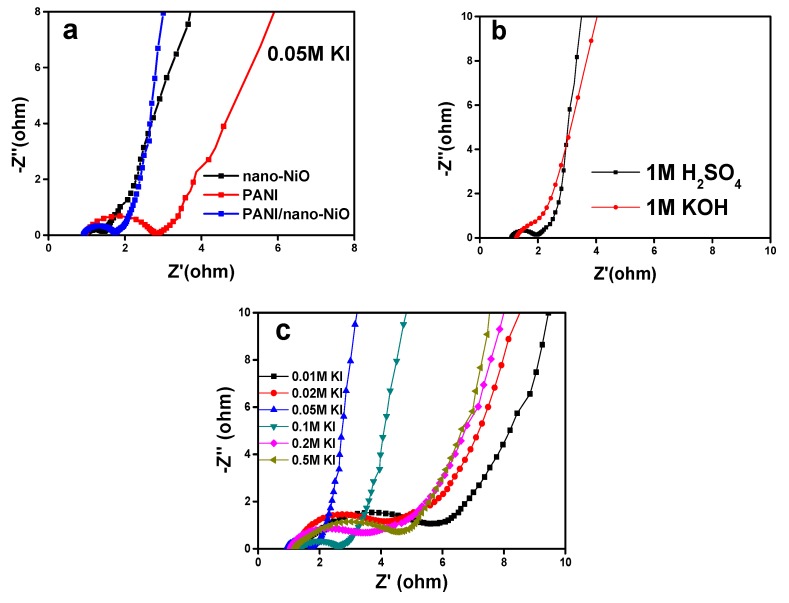
EIS curves in different electrolytes and concentration of KI. (**a**) 0.05 M KI. (**b**) 0 M KI. (**c**) 0.01–0.5 M KI.

Figure 9a presents the EIS of nano-NiO, PANI and PANI/nano-NiO, which can reflect the electron transmission in the bulk of the electrode and the ion diffusion at the interface of electrode and electrolyte. In general, the Nyquist plot consists of a partial semicircle in the high-frequency area and an oblique line at the low-frequency region [41,42]. The negligible semicircle in high-frequency region of PANI/nano-NiO is smaller than that of bulk PANI and nano-NiO, showing low charge transfer and internal resistance. Furthermore, in low-frequency region, a more vertical line, which is more than 45° inclined to the imaginary axis (Z”), suggests a lower diffusive resistance [43]. Figure 9b further rationally explains the better electrochemical reversibility and smaller impedance of PANI/nano-NiO in acid electrolyte, compared to that of in alkaline electrolyte (1 M KOH).

**Figure 10:**
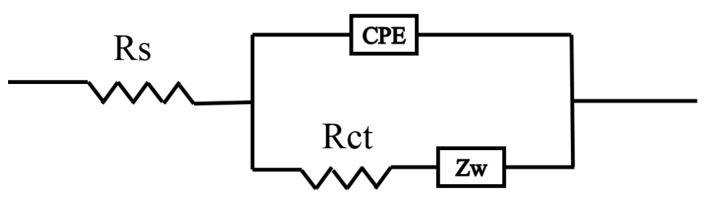
Equivalent circuit model.

The electrochemical impedance spectra of the composites in different concentration of KI range from 0.01 M to 0.5 M are shown in Figure 9c. Figure 10 shows an equivalent circuit model for simulating the capacitance and resistance behavior of PANI/nano-NiO [44]. The electrolyte resistance (Rs, obtained from the intercept on the real impedance axis (Z')) and the charge transfer resistance (Rct, the diameter of the semicircle) are shown in Table 3, as calculated using Zview software (Corrtest Co., Ltd., Wuhan, China).

**Figure 11:**
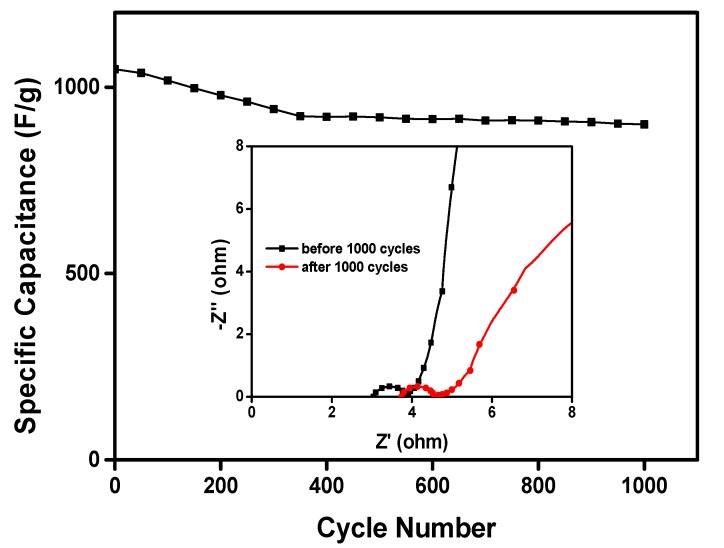
The retention of capacitance after charge–discharge 1000 cycles and the impedance changes.

Figure 11 exhibits the cyclic stability of PANI/nano-NiO carried out at 1.5 A·g^−1^. Noticeably, after 1000 continuous charge–discharge cycles, the specific capacity maintains 948 F·g^−1^ and about 86% retention of its initial specific capacity (1102 F·g^−1^). That is to say, the composite with the good cyclic performance and high rate performance which is well promising for applications in supercapacitors.

## 4. Conclusions

In summary, a new organic/inorganic PANI/nano-NiO nanocomposite electrode material was synthesized for supercapacitors by a special polymerization approach. The nanocomposite possesses a PANI coated structure, leading to interpenetrating organic–inorganic networks which could maximize the synergistic effect of the two components. The composite electrode exhibited an excellent electrochemical performance due to the dual advantages of PANI and nano-NiO. Compared with the alkaline electrolyte, the composite electrode shows a better electrochemical performance in all respects while using the acid electrolyte, proving that the NiO was successfully protected by PANI. By adding the active substance KI, the composite processed a high specific capacitance (2122.75 F·g^−1^ at 0.1 A·g^−1^ in 0.05 M KI electrolyte solution), superior energy density (64.05 Wh·kg^−1^ at 0.2 A·g^−1^ in the two-electrode system) and excellent cycle performance (86% capacitance retention after 1000 cycles at 1.5 A·g^−1^).

## Figures and Tables

**Table 1:** The specific capacitances (F·g^−1^) results.

Sample	Electrolyte	Discharge Time (s)	Current Density (A·g^−1^)	Specific Capacitance (F·g^−1^)
nano-NiO	0.05 M KI	18.5	1	46.25
PANI	0.05 M KI	149	1	372.5
PANI/nano-NiO	0.05 M KI	427.4	1	1068.5
PANI/nano-NiO	1 M KOH	19	0.5	23.75
	1 M H_2_SO_4_	235.9	0.5	235.9
PANI/nano-NiO	0 M KI	1293	0.1	258.6
		632.4	0.2	252.96
		235.9	0.5	235.9
		116.8	1	233.6
		51.2	2	204.8
PANI/nano-NiO	0.05 M KI	8491	0.1	2122.75
		3452.3	0.2	1726.2
		983.6	0.5	1229.5
		427.4	1	1068.5
		83.4	2	417
PANI/nano-NiO	0 M KI	116.8	1	233.6
	0.05 M KI	427.4	1	1068.5
	0.1 M KI	390.1	1	975.25
	0.2 M KI	249.6	1	624
	0.5 M KI	187.4	1	468.5

**Table 2:** Comparison of the specific capacitance and cycle retention.

Materials	Electrolytes	Specific Capacitance (F/g)	Cycle Retention	References
Ni/NiO-3 nanofibers	3M KOH	526 (1 A/g)	79.3% (6000 cycles, 5A/g)	[28]
K_2_Ti_4_O_9_@Ni(OH)_2_/Ti	3 M KOH	340 mF/cm^2^ (50 mV/s)	92.5% (2000 cycles)	[36]
PANI	0.4 M HQ–1 M H_2_SO_4_ (0.5 A/g)	584 (0.5 A/g)	70% (1000 cycles)	[37]
3D RGO-CNI-PANI	1.5 M Li_2_SO_4_	741 (10 mV/s)	-	[38]
PANI/CuO	1 M Na_2_SO_4_ (5 mV/s)	185 (5 mV/s)	75% (2000 cycles)	[39]
PANI/Go	1 M Na_2_SO_4_	596.2 (0.5 A/g)	83.7% (1500 cycles, 2A/g)	[33]
PANI/α-MnMoO_4_	1 M Na_2_SO_4_	396 (5 mV/s)	80.3% (500 cycles)	[40]
PANI/nano-NiO	0.05 M KI–1 M H_2_SO_4_ (0.1 A/g)	2122.75 (0.1 A/g)	86% (1000 cycles, 1.5A/g)	This work

**Table 3:** Rs (Ω) and Rct (Ω) of the PANI/nano-NiO in different concentration of KI.

Sample	Concentration (mol·L^−1^)	Rs (Ω)	Rct (Ω)
PANI/nano-NiO	0.01	1.19	5.21
	0.02	1.26	3.24
	0.05	0.91	1.29
	0.1	1.37	1.64
	0.2	1.05	4.67
	0.5	1.19	2.53
